# Precision of Magnetic Resonance Imaging in Detection of Anterior Cruciate Ligament and Posterior Cruciate Ligament Ruptures and Subsequent Arthroscopic Correlations: An Investigation

**DOI:** 10.1055/s-0044-1785508

**Published:** 2024-12-07

**Authors:** Vijendra Yadav, Chidanand Kaudki Janekunte Chandrashekhar, Rishi Pothuri Ram, Rafeeq Mohammed

**Affiliations:** 1 Sanjay Gandhi Institute of Trauma and Orthopedics, Bangalore, Karnataka, Índia

**Keywords:** anterior cruciate ligament, arthroscopy, knee, magnetic resonance imaging, meniscus

## Abstract

**Objective** The present study endeavors to scrutinize the precision of magnetic resonance imaging as a diagnostic modality for detecting ligament disruption of the knee, with arthroscopy serving as the gold standard. The study delves into the sensitivity, specificity, positive predictive value, and negative predictive value of magnetic resonance imaging (MRI) results in a cohort of 200 patients against diagnostic arthroscopy.

**Methods** Our institution conducted a comprehensive clinical examination of all patients with knee injuries, and those with affirmative findings suggestive of ligament disruption were subjected to an MRI scan. The study comprised 200 patients with MRI-confirmed anterior cruciate ligament (ACL) and posterior cruciate ligament (PCL) tears, who subsequently underwent arthroscopy for both diagnostic and therapeutic purposes. The results were subjected to various statistical tests to compare and analyze the outcomes.

**Results** The study has demonstrated a remarkably high sensitivity and specificity, providing near-optimal accuracy for the diagnosis of ACL and PCL injuries via MRI compared with arthroscopy. Patients with affirmative MRI findings could proceed to undergo diagnostic/therapeutic arthroscopic procedures.

**Conclusion** The study emphasizes the significance of MRI as a noninvasive and highly precise method for assessing ligament injuries in the knee. Although MRI can be used as a first-line investigation, it must be emphasized that arthroscopy remains the gold standard for diagnosing ACL and PCL tears. The current study recommends the use of MRI as a valuable screening tool in patients with suspected knee ligament disruption, with potential to reduce the number of diagnostic arthroscopies in patients with inconclusive clinical findings, thereby minimizing patient discomfort and healthcare costs.

## Introduction

In contemporary times, injuries to the knee joint resulting from road traffic accidents and sports activities are ubiquitous. 1 These injuries typically involve the ligaments and meniscus of the knee, disrupting the stability and normal biomechanics of the joint and hindering routine daily activity. Hence, prompt and precise diagnosis and management of such injuries are imperative. Among the common knee injuries are anterior cruciate ligament (ACL) or combined ACL and posterior cruciate ligament (PCL) injuries. Initially, in the late 1960s and early 1970s, orthopedic surgeons solely relied on clinical examination until numerous reports suggested the efficacy of arthroscopy in diagnosing and treating various knee disorders. 2 The advent of magnetic resonance imaging (MRI) has revolutionized the diagnosis and management of ACL and meniscal tears of the knee, making arthroscopy less necessary. This study aims to evaluate the radiological and arthroscopic findings of the anterior and posterior cruciate ligaments, correlate the outcomes of both techniques, and determine which method is superior in accurately diagnosing ACL and PCL injuries.

## Methods

The study was approved by the institutional ethics committee number ECR/1092/Inst/KA/2018.

A prospective study was undertaken on a cohort of 200 individuals who were admitted to a tertiary health care center with knee injuries arising from diverse etiologies and who satisfied the inclusion criteria. The study was conducted over a period spanning from November 2019 to November 2022.

### Inclusion Criteria

1. Patients aged between 18 and 60 years.
2. Patients with positive MRI findings indicating ACL, PCL, or both injuries.
3. Patients who subsequently underwent arthroscopy for further evaluation and treatment.
4. Patients with confirmed ACL, PCL, or both injuries based on arthroscopic examination, utilizing the routine method described below.

### Routine Arthroscopy Method

The routine arthroscopy method involved the use of anteromedial and lateral portals. Careful probing of the posterior cruciate ligament (PCL) was conducted to assess for laxity or lesions. In cases in which PCL issues were suspected based on clinical examination or preoperative MRI scans, the posteromedial portal was consistently employed during arthroscopic examination to confirm these findings and ensure a comprehensive evaluation of the PCL. The utilization of the posteromedial portal allowed for a more thorough assessment and characterization of PCL injuries identified through clinical examination or preoperative imaging.

By incorporating the routine arthroscopy method into the inclusion criteria, the paper provides clarity on the methodology used in the study, ensuring that patients with positive MRI findings underwent arthroscopy following a specific routine that includes the use of the anteromedial and lateral portals, as well as the potential use of the posteromedial portal for comprehensive evaluation of PCL injuries.

### Exclusion Criteria

1. Patients with contraindications to MRI, such as having intracerebral aneurysmal clips, cardiac pacemakers, metallic foreign bodies in the eye, or implants in the middle ear.
2. Patients who have had a recent knee injury but, upon clinical examination, exhibit no instability.
3. Patients who are deemed unfit for anesthesia.

## Methodology

Following the elicitation of medical history, a comprehensive clinical examination was conducted, wherein various tests were performed to assess the extent of knee injury. Specifically, the Lachman and anterior drawer tests were employed to evaluate the anterior cruciate ligament (ACL) injury, whereas the Godfrey sag sign and posterior drawer tests were employed to evaluate the posterior cruciate ligament (PCL) injuries.

Magnetic resonance imaging (MRI) was then performed using the 1.5 Tesla MR protocol on a General Electric Healthcare Company (GE Signa Voyager 1.5T, Waukesha, Wisconsin, USA) 1.5 T MRI machine. T1 and T2 weighted sequences were performed on axial, coronal, and sagittal planes. The MR films were meticulously assessed by a highly skilled radiologist, who carefully documented the status of the cruciate ligaments.

Subsequently, arthroscopic surgery was performed under spinal anesthesia, with the patient placed in a supine position, with lateral support to the proximal thigh. A proximal thigh tourniquet was utilized for each case. The operating surgeon was not apprised of the MRI findings.

The acquired data were analyzed using the sophisticated statistical tool SPSS software Statistics 28.0, IBM Corporation (Armonk, New York, USA). Continuous variables were analyzed by computing the sensitivity, specificity, positive predictive value (PPV), and negative predictive value (NPV). A *P* -value lower than 0.05 was deemed statistically significant.

To ascertain the sensitivity, specificity, and accuracy of magnetic resonance imaging (MRI), the outcomes obtained through arthroscopic examination were considered to represent the true diagnosis. Sensitivity, which represents the ability of the MRI to identify individuals with the condition, was computed as the ratio of true positive results to the sum of true positive and false negative results.

Specificity, which reflects the MRI's capacity to accurately identify those without the condition, was determined as the ratio of true negative results to the sum of true negative and false positive results.

Accuracy, which represents the overall ability of the MRI to identify both positive and negative cases correctly, was calculated as the summation of true positive and true negative results divided by the total number of patients who underwent arthroscopy.

The combined data was meticulously compiled and categorized into four distinct groups, based on their correlation with the MRI findings. These categories included the following:

First, the true-positive result signified instances in which the MRI diagnosis was accurately confirmed by arthroscopy.

Second, the true-negative result, which pertained to cases in which the MRI yielded a negative finding for the lesion, and this was corroborated by the arthroscopic examination.

Third, the false-positive result, which represented situations in which the MRI indicated the presence of a lesion, but arthroscopy failed to confirm its existence.

Lastly, the false-negative result, which referred to cases in which arthroscopy detected the presence of the lesion, but the MRI failed to reveal any indication of it.

## Results

A total of 200 patients who exhibited traumatic ACL and PCL injuries were carefully identified and examined through a retrospective and prospective approach, wherein MRI evaluation was followed by arthroscopic surgery. Specifically, patients who demonstrated suspected ACL and PCL injuries and fulfilled the predetermined inclusion criteria were carefully selected for inclusion in the study.

Notably, individuals who displayed degenerative changes or evidence of loose bodies in plain radiographs, those who were deemed unfit for anesthesia, and those who had undergone non-operative treatments were all carefully excluded from the study to ensure its validity.

The collected data was meticulously analyzed to determine the true positives, true negatives, false positives, and false negatives associated with the study. Using specificity and sensitivity measures, PPV and NPV were then calculated with the aid of arthroscopic examination, which served as the gold standard for comparison.

### Age Distribution

This study was conducted on patients aged 18 to 60, with a mean age of 35.7 years at admission.

The highest frequency and percentage are observed in the 20-to-24 age group with 60 individuals, which accounts for 30% of the total sample. The remaining age groups range from 7 to 13% in frequency and 4 to 6% in percentage.

### Mode of Injury

Road traffic accidents were our study's most common mode of injury, accounting for ∼ 60%.

### Anterior Cruciate Ligament

Sensitivity: TP / (TP + FN) = 138 / (138 + 16) = 0.896 or 89.6%

Specificity: TN / (TN + FP) = 37 / (37 + 9) = 0.804 or 80.4%

PPV: TP / (TP + FP) = 138 / (138 + 9) = 0.939 or 93.9%

NPV: TN / (TN + FN) = 37 / (37 + 16) = 0.698 or 69.8%

Accuracy: (TP + TN) / (TP + TN + FP + FN) = (138 + 37) / 200 = 0.875 or 87.5%

Therefore, the MRI test has a high sensitivity (89.6%), indicating that it correctly identifies the majority of arthroscopically positive cases, but a lower specificity (80.4%), indicating that it also identifies some arthroscopically negative cases as positive. The PPV of the test is high (93.9%), meaning that if the MRI test is positive, there is a high probability that the patient is truly arthroscopically positive. However, the NPV is lower (69.8%), indicating that if the MRI test is negative, there is still a significant probability that the patient is arthroscopically positive. Overall, the MRI test has an accuracy of 87.5%, which is relatively good but not perfect.

### Posterior Cruciate Ligament

Sensitivity: TP / (TP + FN) = 45 / (45 + 15) = 0.75 or 75%

Specificity: TN / (TN + FP) = 123 / (123 + 17) = 0.878 or 87.8%

PPV: TP / (TP + FP) = 45 / (45 + 17) = 0.726 or 72.6%

NPV: TN / (TN + FN) = 123 / (123 + 15) = 0.891 or 89.1%

Accuracy: (TP + TN) / (TP + TN + FP + FN) = (45 + 123) / 200 = 0.84 or 84%

Therefore, the MRI test has a sensitivity of 75%, indicating that it correctly identifies 75% of arthroscopically positive cases. The specificity is higher at 87.8%, indicating that it correctly identifies a higher proportion of arthroscopically negative cases. The PPV is 72.6%, meaning that if the MRI test is positive, there is a 72.6% probability that the patient is truly arthroscopically positive. The NPV is higher at 89.1%, indicating that if the MRI test is negative, there is a higher probability that the patient is truly arthroscopically negative. Overall, the MRI test has an accuracy of 84%, which is moderately good but not perfect.

## Discussion

The knee joint's MRI scan is considered an efficacious non-invasive diagnostic tool and a preferred alternative to diagnostic arthroscopy. In current clinical practice, MRI scans are routinely performed to confirm the diagnosis of anterior cruciate ligament (ACL) and posterior cruciate ligament (PCL) injuries. Nonetheless, discerning partial tears may pose challenges and vary based on the observer and the scanner's sensitivity.

The current study aims to compare and correlate the MRI and arthroscopic findings in diagnosing ACL and PCL injuries. The study cohort encompassed individuals aged 18 to 60 years, with the youngest male patient being 18 years old, and the oldest female being 60 years old. Males were more prone to knee injuries and underwent surgery at a younger age, primarily due to their active involvement in sports. Furthermore, the right knee was more commonly injured than the left. 3

Prior studies have reported high sensitivity and specificity in diagnosing ACL tears using MRI scans. Rubin et al. 4 reported 93% sensitivity for diagnosing isolated ACL tears. A sensitivity of 92 to 100% and specificity of 93–100% for the MR imaging diagnosis of ACL tears has been reported by similar studies in the past. 5 The current study attested to a sensitivity of 90.90% and a specificity of 78.26% for MRI in diagnosing ACL tears, displaying a fair correlation with arthroscopy. The accuracy of MRI in detecting ACL tears was 88%, categorizing it in the “very good” interpretation group (80–90%). The results were consistent with prior literature, suggesting an 80-to-94% accuracy range in detecting crucial ligament tears. The PPV of MRI was 93.33%, whereas the NPV was 72%.

The interpretation of MRI results is highly reliant on the experience and training of the radiologist. 6 7 8 Arthroscopy serves as the reference point in most knee MRI studies owing to its technical demands, and the results are subject to the surgeon's experience, primarily in challenging cases. Magnetic resonance imaging remains the quintessential diagnostic tool for ACL and PCL injuries, with reported accuracy ranging from 70 to 100%. 9 However, arthroscopy should be considered an adjunct to a comprehensive clinical examination, including a thorough history, physical examination, and appropriate radiographs. Surgical alternatives are thoroughly discussed with the patient before the procedure, and the definitive surgical procedure is performed during an arthroscopic examination.

Several studies have validated the utility of MRI scans in diagnosing ACL and PCL injuries. Despite the observer variability in interpreting MRI results, it remains an indispensable diagnostic tool in current clinical practice. 10 Arthroscopy, however, should be used in conjunction with a comprehensive clinical examination, providing a definitive diagnosis and treatment plan for patients.

## Conclusion

Our research has conclusively demonstrated that MRI is an exceedingly reliable tool in the diagnosis of ACL and PCL injuries in the knee joint. The sensitivity, specificity, and overall accuracy of MRI are exceptionally high, thus affirming its indispensable role in identifying such injuries. As such, it is an ideal screening tool, rendering diagnostic arthroscopy unnecessary for diagnosis in the vast majority of patients.

Notably, MRI is both accurate and non-invasive, making it an optimal modality for assessing ligamentous injuries. In light of these findings, we conclude that MRI should be the first-line investigation for patients presenting with a knee injury, in whom ligamentous injury is suspected.

## References

[1] Kaplan P A Walker C W Kilcoyne R F Brown D E Tusek D Dussault R G Occult fracture patterns of the knee associated with anterior cruciate ligament tears: assessment with MR imaging Radiology 1992 183 03 835 838 1584943 10.1148/radiology.183.3.1584943

[2] Levine W N Bergfeld J A Tessendorf W Moorman C T Intracapsular and extracapsular structure relationships at the knee: magnetic resonance imaging and arthroscopic correlation Arthroscopy 1997 13 02 166 17079127073

[3] Avcu S Altun E Akpinar I Bulut M D Eresov K Biren T Knee joint examinations by magnetic resonance imaging: The correlation of pathology, age, and sex N Am J Med Sci 2010 2 04 202 204 22624141 10.4297/najms.2010.2202 PMC3354411

[4] Rubin D A Kettering J M Towers J D Britton C A MR imaging of knees having isolated and combined ligament injuries AJR Am J Roentgenol 1998 170 05 1207 1213 9574586 10.2214/ajr.170.5.9574586

[5] Lee K Seigel M J Lau D M Hildebolt C F Matava M J Anterior cruciate ligament tears: MR imaging-based diagnosis in a pediatric population Radiology 1999 213 03 697 704 10580941 10.1148/radiology.213.3.r99dc26697

[6] Oei E H Nikken J J Verstijnen A C Ginai A Z Myriam Hunink M G MR imaging of the menisci and cruciate ligaments: a systematic review Radiology 2003 226 03 837 848 12601211 10.1148/radiol.2263011892

[7] Esmaili Jah A A Keyhani S Zarei R Moghaddam A K Accuracy of MRI in comparison with clinical and arthroscopic findings in ligamentous and meniscal injuries of the knee Acta Orthop Belg 2005 71 02 189 196 16152853

[8] Navali A M Bazavar M Mohseni M A Safari B Tabrizi A Arthroscopic evaluation of the accuracy of clinical examination versus MRI in diagnosing meniscus tears and cruciate ligament ruptures Arch Iran Med 2013 16 04 229 232 23496367

[9] Li K Du J Huang L X Ni L Liu T Yang H L The diagnostic accuracy of magnetic resonance imaging for anterior cruciate ligament injury in comparison to arthroscopy: a meta-analysis Sci Rep 2017 7 01 7583 28790406 10.1038/s41598-017-08133-4 PMC5548790

[10] Shantanu K Singh S Srivastava S Saroj A K The Validation of Clinical Examination and MRI as a Diagnostic Tool for Cruciate Ligaments and Meniscus Injuries of the Knee Against Diagnostic Arthroscopy Cureus 2021 13 06 e15727 34290922 10.7759/cureus.15727 PMC8289396

