# Supplementary material for: Precision of Magnetic Resonance Imaging in Detection of Anterior Cruciate Ligament and Posterior Cruciate Ligament Ruptures and Subsequent Arthroscopic Correlations: An Investigation
Source: Rev Bras Ortop (Sao Paulo). 2024 Dec 7;59(5):e707–11. doi: 10.1055/s-0044-1785508 (PMC11624920; doi:10.1055/s-0044-1785508)
Supplement: Related texts

# Research article: Precisão da ressonância magnética na detecção de rupturas do ligamento cruzado anterior e do ligamento cruzado posterior e correlações artroscópicas subsequentes: Uma investigação

**Keywords:** artroscopia, imagem de ressonância magnética, joelho, ligamento cruzado anterior, menisco

## Abstract

**Objetivo** Este estudo examinou a precisão da ressonância magnética (RM) como modalidade diagnóstica para detecção da ruptura de ligamentos do joelho, em que a artroscopia é o padrão-ouro. O estudo investigou a sensibilidade, especificidade, valor preditivo positivo e valor preditivo negativo dos resultados da RM em uma coorte de 200 pacientes em comparação à artroscopia diagnóstica.

**Métodos** Nossa instituição realizou um exame clínico abrangente de todos os pacientes com lesões no joelho e aqueles com achados afirmativos sugestivos de ruptura ligamentar foram submetidos à RM. O estudo incluiu 200 pacientes com rupturas do ligamento cruzado anterior (LCA) e do ligamento cruzado posterior (LCP) confirmadas por RM que, posteriormente, foram submetidos à artroscopia para fins diagnósticos e terapêuticos. Os resultados foram submetidos a vários testes estatísticos para comparação e análise dos desfechos.

**Resultados** O estudo demonstrou sensibilidade e especificidade notavelmente altas, com acurácia quase ideal para o diagnóstico de lesões do LCA e do LCP por meio de RM em comparação à artroscopia. Os pacientes com resultados afirmativos à RM poderiam ser submetidos a procedimentos artroscópicos diagnósticos ou terapêuticos.

**Conclusão** O estudo enfatiza a importância da RM como método não invasivo e altamente preciso para avaliação das lesões ligamentares do joelho. Embora a RM possa ser usada na investigação de primeira linha, deve-se enfatizar que a artroscopia continua a ser o padrão-ouro para o diagnóstico de rupturas do LCA e do LCP. Este estudo recomenda o uso da RM como uma valiosa ferramenta de triagem em pacientes com suspeita de ruptura de ligamentos do joelho, com potencial para reduzir o número de artroscopias diagnósticas em indivíduos com achados clínicos inconclusivos, o que minimiza o desconforto do paciente e os custos de saúde.

## Introdução

Hoje, as lesões na articulação do joelho resultantes de acidentes de trânsito e atividades esportivas são onipresentes. 1 Essas lesões geralmente acometem os ligamentos e o menisco do joelho, alterando a estabilidade e a biomecânica normal da articulação e dificultando as atividades diárias rotineiras. Portanto, o diagnóstico e o manejo rápido e preciso de tais lesões são imperativos. Entre as lesões comuns do joelho estão aquelas que afetam o ligamento cruzado anterior (LCA) e/ou o ligamento cruzado posterior (LCP). A princípio, entre o final da década de 1960 e o início da década de 1970, os cirurgiões ortopédicos baseavam-se apenas no exame clínico, até que numerosos relatos sugeriram a eficácia da artroscopia no diagnóstico e tratamento de várias doenças dessa articulação. 2 O advento da ressonância magnética (RM) revolucionou o diagnóstico e o tratamento das rupturas de LCA e menisco, diminuindo a necessidade de artroscopia. Este estudo teve como objetivo avaliar os achados radiológicos e artroscópicos do LCA e LCP, correlacionar os resultados de ambas as técnicas e determinar qual método é superior no diagnóstico preciso das lesões destes ligamentos.

## Métodos

Este estudo foi aprovado pelo Comitê de Ética institucional sob número ECR/1092/Inst/KA/2018.

Foi realizado um estudo prospectivo em uma coorte de 200 indivíduos internados em um centro de saúde terciário com lesões no joelho decorrentes de diversas etiologias e que satisfizeram os critérios de inclusão. O estudo foi conduzido entre novembro de 2019 e novembro de 2022.

## Critérios de inclusão

1. Pacientes entre 18 e 60 anos de idade.
2. Pacientes com resultados positivos à RM indicando lesões de LCA e/ou LCP.
3. Pacientes posteriormente submetidos à artroscopia para avaliação e tratamento adicionais.
4. Pacientes com lesões confirmadas de LCA e/ou LCP com base em exame artroscópico utilizando o método de rotina descrito a seguir.

## Método artroscópico de rotina

O método artroscópico de rotina usou portais anteromedial e lateral. A sondagem cuidadosa do LCP avaliou a presença de lassidão ou lesões. Nos casos com suspeita de lesões do LCP com base no exame clínico ou na RM pré-operatória, o portal posteromedial foi empregado consistentemente durante o exame artroscópico para confirmar esses achados e garantir a análise abrangente do LCP. A utilização do portal posteromedial permitiu a avaliação e caracterização mais completas das lesões do LCP identificadas por meio de exame clínico ou imagem pré-operatória.

Ao incorporar o método artroscópico de rotina nos critérios de inclusão, o artigo esclarece a metodologia utilizada no estudo, assegurando que os pacientes com resultados positivos à RM fossem submetidos à artroscopia seguindo um procedimento específico que inclui o uso de portais anteromediais e laterais, bem como o possível uso do portal posteromedial para avaliação abrangente de lesões do LCP.

## Critérios de exclusão

1. Pacientes com contraindicações para RM, como clipes de aneurisma intracerebral, marcapassos cardíacos, corpos estranhos metálicos no olho ou implantes na orelha média.
2. Pacientes com lesão recente no joelho, mas que, ao exame clínico, não apresentavam instabilidade.
3. Pacientes considerados maus candidatos à anestesia.

## Metodologia

Após a obtenção dos prontuários médicos, foi realizado um exame clínico abrangente, com diversos testes para avaliação da extensão da lesão no joelho. Especificamente, os testes de Lachman e de gaveta anterior foram empregados para análise da lesão do LCA, enquanto o sinal de afundamento de Godfrey e os testes de gaveta posterior foram empregados para avaliação das lesões do LCP.

A RM foi então realizada usando o protocolo de 1,5 Tesla em um equipamento de 1,5 T da General Electric Healthcare Company (GE Signa Voyager 1.5T, Waukesha, Wisconsin, EUA). As sequências ponderadas em T1 e T2 foram obtidas nos planos axial, coronal e sagital. As imagens foram avaliadas de forma meticulosa por um radiologista altamente qualificado, que documentou cuidadosamente o estado dos ligamentos cruzados.

Depois, foi realizada cirurgia artroscópica sob raquianestesia com o paciente posicionado em decúbito dorsal e apoio lateral na coxa proximal. Um torniquete proximal na coxa foi utilizado para cada caso. O cirurgião não foi informado dos resultados da RM.

Os dados adquiridos foram analisados com a sofisticada ferramenta estatística do *software* SPSS Statistics 28.0, IBM Corporation (Armonk, New York, EUA). As variáveis contínuas foram analisadas calculando-se a sensibilidade, a especificidade, o valor preditivo positivo (VPP) e o valor preditivo negativo (VPN). O valor de *p* inferior a 0,05 foi considerado estatisticamente significativo.

Para verificar a sensibilidade, especificidade e acurácia da RM, os resultados do exame artroscópico foram considerados representativos do verdadeiro diagnóstico. A sensibilidade, que representa a capacidade da RM de identificar indivíduos com a doença, foi calculada como a razão entre resultados verdadeiro-positivos (VP) e a soma de resultados VP e falso-negativos (FN).

A especificidade, que reflete a capacidade da RM de identificar com acurácia aqueles sem a doença, foi determinada como a razão entre resultados verdadeiro-negativos (VN) e a soma de resultados VN e falso-positivos (FP).

A acurácia, que representa a capacidade geral da RM de identificar corretamente os casos positivos e negativos, foi calculada como a soma dos resultados VP e VN dividida pelo número total de pacientes submetidos à artroscopia.

Os dados combinados foram meticulosamente compilados e categorizados em quatro grupos distintos, com base na sua correlação com os resultados RM. Essas categorias foram as seguintes:

Na primeira categoria, o resultado VP indicou casos em que o diagnóstico à RM foi confirmado com acurácia pela artroscopia.

Na segunda, o resultado VN, referente aos casos em que a RM foi negativa para a lesão, o que foi corroborado pelo exame artroscópico.

Na terceira, o resultado FP, que representou situações em que a RM indicava a presença de lesão, mas a artroscopia não conseguia confirmar sua existência.

Na quarta e última categoria, o resultado FN, que se referia aos casos em que a artroscopia detectou a presença da lesão, mas a RM não revelou qualquer indício dela.

## Resultados

Um total de 200 pacientes com lesões traumáticas do LCA e LCP foram cuidadosamente identificados e examinados por meio de uma abordagem retrospectiva e prospectiva em que a avaliação por RM foi seguida por cirurgia artroscópica. Especificamente, os pacientes com suspeita de lesão do LCA ou LCP que atenderam aos critérios de inclusão pré-determinados foram cuidadosamente selecionados para inclusão no estudo.

Notavelmente, os indivíduos com alterações degenerativas ou evidências de corpos livres em radiografias simples, aqueles que foram considerados maus candidatos à anestesia e aqueles que foram submetidos a tratamentos não cirúrgicos foram cuidadosamente excluídos do estudo para assegurar sua validade.

Os dados coletados foram analisados meticulosamente para determinar os resultados VP, VN, FP e FN associados ao estudo. Por meio da utilização de medidas de especificidade e sensibilidade, foram calculados o VPP e o VPN com auxílio do exame artroscópico, que foi o padrão-ouro para comparação.

## Distribuição etária

Este estudo foi realizado em pacientes com idade entre 18 e 60 anos, com idade média de 35,7 anos à admissão.

As maiores frequência e percentual foram observados na faixa etária de 20 a 24 anos com 60 indivíduos, o que representa 30% do total da amostra. As restantes faixas etárias variam entre 7 e 13% em frequência e 4 e 6% em percentagem.

## Modo de lesão

O acidente de trânsito foi o modo de lesão mais comum em nosso estudo, representando cerca de 60% dos casos.

## Ligamento cruzado anterior

Sensibilidade: VP / (VP + FN) = 138 / (138 + 16) = 0,896 ou 89,6%

Especificidade: VN / (VN + FP) = 37 / (37 + 9) = 0,804 ou 80,4%

VPP: VP / (VP + FP) = 138 / (138 + 9) = 0,939 ou 93,9%

VPN: VN / (VN + FN) = 37 / (37 + 16) = 0,698 ou 69,8%

Acurácia: (VP + VN) / (VP + VN + FP + FN) = (138 + 37) / 200 = 0,875 ou 87,5%

Portanto, o exame de RM tem alta sensibilidade (89,6%), indicando que identifica corretamente a maioria dos casos artroscopicamente positivos, mas uma especificidade menor (80,4%), mostrando que também identifica alguns casos artroscopicamente negativos como positivos. O VPP do teste é alto (93,9%), o que significa que se o exame de RM for positivo, há uma grande probabilidade de o paciente ser verdadeiramente positivo à artroscopia. Porém, o VPN é menor (69,8%), indicando que se a RM for negativa, ainda há uma probabilidade significativa de o paciente ser artroscopicamente positivo. De modo geral, a RM tem acurácia de 87,5%, um valor relativamente bom, mas não perfeito.

## Ligamento cruzado posterior

Sensibilidade: VP / (VP + FN) = 45 / (45 + 15) = 0,75 ou 75%

Especificidade: VN / (VN + FP) = 123 / (123 + 17) = 0,878 ou 87,8%

VPN: VN / (VN + FN) = 45 / (45 + 17) = 0,726 ou 72,6%

VPN: VN / (VN + FN) = 123 / (123 + 15) = 0,891 ou 89,1%

Acurácia: (VP + VN) / (VP + VN + FP + FN) = (45 + 123) / 200 = 0,84 ou 84%

Portanto, a RM tem sensibilidade de 75%, indicando que identifica corretamente 75% dos casos artroscopicamente positivos. A especificidade é maior, de 87,8%, indicando que identifica corretamente uma proporção maior de casos artroscopicamente negativos. O VPP é de 72,6%, indicando que se a RM for positiva, há 72,6% de probabilidade de o paciente ser verdadeiramente positivo à artroscopia. O VPN é maior, de 89,1%, indicando que se a RM for negativa, há maior probabilidade de o paciente ser realmente negativo à artroscopia. De modo geral, a RM tem acurácia de 84%, um valor moderadamente bom, mas não perfeito.

## Discussão

A RM da articulação do joelho é considerada uma ferramenta diagnóstica não invasiva eficaz e uma alternativa preferida à artroscopia diagnóstica. Na prática clínica atual, a RM é realizada rotineiramente para confirmar o diagnóstico de lesões do LCA e LCP. No entanto, discernir rupturas parciais pode representar desafios e variar dependendo do observador e da sensibilidade do equipamento.

Este estudo teve como objetivo comparar e correlacionar os achados de RM e artroscópicos no diagnóstico de lesões do LCA e LCP. A coorte do estudo abrangeu indivíduos com idade entre 18 e 60 anos, sendo o paciente mais jovem do sexo masculino com 18 anos e o paciente mais velho do sexo feminino com 60 anos. Os homens eram mais propensos a lesões nos joelhos e foram submetidos a cirurgias em idades menores, principalmente devido ao seu envolvimento ativo em esportes. Além disso, o joelho direito foi mais comumente acometido do que o esquerdo. 3

Estudos anteriores relataram alta sensibilidade e especificidade no diagnóstico de rupturas do LCA usando exames de RM. Rubin et al. 4 relataram sensibilidade de 93% para o diagnóstico de rupturas isoladas do LCA. Uma sensibilidade entre 92 e 100% e especificidade entre 93 e 100% foi observada para o diagnóstico por RM de rupturas do LCA demonstradas por estudos semelhantes no passado. 5 Nosso estudo observou uma sensibilidade de 90,90% e uma especificidade de 78,26% para RM em diagnosticar rupturas do LCA, apresentando uma boa correlação com a artroscopia. A acurácia da RM na detecção de rupturas do LCA foi de 88%, categorizando-a no grupo de interpretação “muito boa” (80–90%). Os resultados foram consistentes com a literatura, sugerindo uma faixa de acurácia de 80 a 94% na detecção de rupturas de ligamentos cruzados. O VPP da RM foi de 93,33%, enquanto o VPN foi de 72%.

A interpretação dos resultados da RM depende muito da experiência e do treinamento do radiologista. 6 7 8 A artroscopia é uma referência na maioria dos estudos de RM do joelho devido às suas demandas técnicas, e os resultados estão sujeitos à experiência do cirurgião, principalmente em casos desafiadores. A RM continua a ser a ferramenta diagnóstica por excelência para lesões do LCA e do LCP, com acurácia relatada entre 70 e 100%. 9 No entanto, a artroscopia deve ser considerada um complemento de um exame clínico abrangente, incluindo anamnese completa, exame físico e radiografias apropriadas. As alternativas cirúrgicas são discutidas minuciosamente com o paciente antes do procedimento, e o procedimento cirúrgico definitivo ocorre durante o exame artroscópico.

Diversos estudos validaram a utilidade da RM no diagnóstico de lesões do LCA e LCP. Apesar da variabilidade do observador na interpretação dos resultados da RM, esta técnica ainda é uma ferramenta diagnóstica indispensável na prática clínica atual. 10 A artroscopia, entretanto, deve ser usada em conjunto com um exame clínico abrangente, fornecendo um diagnóstico definitivo e um plano de tratamento para os pacientes.

## Conclusão

Nosso estudo demonstrou conclusivamente que a RM é uma ferramenta extremamente confiável no diagnóstico de lesões do LCA e do LCP na articulação do joelho. A sensibilidade, especificidade e acurácia global da RM são excepcionalmente altas, afirmando assim o seu papel indispensável na identificação de tais lesões. Como tal, é uma ferramenta de rastreio ideal, tornando a artroscopia diagnóstica desnecessária para o diagnóstico na grande maioria dos pacientes.

Notavelmente, a RM é precisa e não invasiva, tornando-a uma modalidade ideal para avaliação de lesões ligamentares. À luz destes achados, concluímos que a RM deve ser a investigação de primeira linha em pacientes com lesão no joelho e suspeita de acometimento ligamentar.
